# Eating for Strength

**Published:** 1890-02

**Authors:** 


### Eating for Strength; or, Food and Diet in their Relation to Health and Work, together with several hundred recipes for Wholesome Food and Drinks. By M. L. Holbrook, M. D., Professor of Hygiene in the New York Medical College and Hospital for Women, etc. New York: M. L. Holbrook & Co.

The title is sufficient to indicate the scope of this work. Its contents bear out the title. It is an excellent work on the subject, and should be in the hands of not only physicians, but all others as well. It will be found useful each day and every day.

